# Does Medical Curriculum Impact on Empathy? A Longitudinal Study in a Sample of Undergraduate Medical Students

**DOI:** 10.1007/s40670-024-02053-5

**Published:** 2024-04-25

**Authors:** Stefano Ardenghi, Selena Russo, Giulia Rampoldi, Marco Bani, Maria Grazia Strepparava

**Affiliations:** 1 School of Medicine and Surgery, University of Milano-Bicocca, Via Cadore 48, 20900 Monza, MB Italy; 2 Fondazione I.R.C.C.S. San Gerardo dei Tintori, Via Pergolesi 33, 20900 Monza, MB Italy

**Keywords:** Empathy, Medical students, Medical education, Gender differences, Longitudinal

## Abstract

Empathy in medical students is receiving increasing attention as it is fundamental to build and develop a functional patient-physician relationship. When looking at its determinants, demographic and academic factors seem to concur in shaping empathy in this population. Although data show strong gender differences and changes in empathy throughout medical school, it is not clear the direction of these changes and whether gender and curriculum features modulate them. This longitudinal study examined changes in empathy and explored gender differences throughout the medical school. Four consecutive cohorts of Italian medical students (*N* = 336) completed the Jefferson Scale of Empathy – Student (JSE-S) and the Interpersonal Reactivity Index (IRI) in their second year of study (before any clinical clerkship and communication skills courses) and fifth year of study (after a 2-year clinical clerkship and communication skills courses). Analysis of variance for repeated-measures revealed that, beyond the effect of gender, JSE-S total score and IRI Perspective Taking increased, whereas IRI Personal Distress and IRI Fantasy significantly decreased throughout medical school. No significant change in IRI Empathic Concern emerged over time. Student’s *t*-tests showed that female students displayed significantly higher mean scores than their male counterparts for all empathy measures in both their second and fifth years of medical training. The findings suggest that the medical curriculum affects self-reported empathy dimensions among undergraduate medical students. Further research is needed to deepen the understanding of the educational factors that promote the changes in empathy levels during medical training.

## Introduction

Empathy is fundamental for building and developing a functional patient-physician relationship [1]. Different definitions of empathy are available. According to Davis’s conceptualization [2], empathy is a multidimensional construct including four dimensions. Two are cognitive dimensions and relate to spontaneously adopting the psychological point of view of others and to transpose oneself imaginatively into the feelings and actions of fictitious characters, whereas the other two are emotional dimensions and refer to feel sympathy and concern for unfortunate others and to feel unease in tense interpersonal settings. Although both components of empathy are regarded as immensely valuable by health training entities [3], the cognitive one is the most studied when it comes to the patient-physician relationship as the ability to understand the patient’s feelings without transposing into the patient’s situation is considered not to impair physicians’ professionalism [4]. In their pioneering work on empathy in healthcare settings, Hojat et al. [5] described empathy as a predominantly cognitive construct entailing understanding other people’s disease-related feelings and experiences as well as communicating this understanding and openness to help.

Because of an empathetic relationship, patients are encouraged to share precise and relevant clinical information allowing for better diagnostics and clinical outcomes [6, 7]. Patient satisfaction, compliance, and patient empowerment are the three key outcomes of empathic communication [8–10]. Empathy is also a protective factor for physicians and medical students against burnout syndrome and it is strictly connected to professional satisfaction [11–13]. Considering all the positive outcomes related to an empathic approach in medicine, it is of paramount importance to further our understanding of the determinants of empathy in future healthcare providers and how to promote it. It is therefore not surprising the growing research interest worldwide around this topic [14]. Medical students’ empathy has been positively associated with psycho-attitudinal variables, such as secure attachment style [15], ability to regulate emotions [16], patient-centeredness [17, 18], dispositional mindfulness [19–21], self-transcending personal values [22], adaptive coping strategies [23], and self-efficacy and some of the Big Five personality traits [24]. As for the role of gender, it seems to be a strong cross-cultural predictor of empathic orientation among healthcare students and professionals [25]. During patient-physician interactions, female physicians focus more on psycho-social factors and manage the relationship in a more patient-centered way than males [26]. It has been advanced that this gender difference lies in a sort of self-fulfilling prophecy of Western cultures where females are traditionally expected to be aware of and take care of others’ feelings and emotions more than males [25, 27].

The role of the medical education path is not clear-cut and the debate on the trajectories and intensity of empathy changes throughout medical school is still ongoing. While some studies have found a significant decline in self-reported empathy in medical students during their studies [28–32], others have shown an increase [33–37] or stability in empathy levels [38–45]. The inconsistency of these findings was related to the different characteristics of medical curricula: medical school programs that focus more on hard and technical skills with less emphasis on communication and soft skills often report a decline in students’ empathy [46]. The structure and content of medical curricula, furthermore, are strongly linked to national regulations as well as cultural factors. Here, the need for investigations in different countries will allow comparing and unveiling the role of cultural- and educational-related factors in shaping empathy in healthcare students. This data is crucial to develop tailored and successful educational programs aiming to foster empathy in the tomorrow’s physicians. Such investigations are missing and strongly needed in the Italian context.

This longitudinal study aimed to further our understanding of the relationship between empathy and medical education while exploring gender role in a sample of undergraduate medical students in Italy. We hypothesized that medical students experience an increase in empathy levels during their studies, with female students showing higher levels of empathy compared to males throughout medical school.

## Methods

### The Medical School Program at the Study Center

The degree program in Medicine and Surgery in Italy is 6 year-long with all national curricula aligned to the recommendations of the Permanent Conference of Italian of Medical School Directors. The organization of the academic activities and their contents are similar throughout the country with the first 2 years of medical school being considered pre-clinical and with minimal interaction with patients, while the remaining 4 years provide students with clinical clerkship experiences. The curricula of the study center, that is, the Milano-Bicocca School of Medicine and Surgery (Italy), is characterized by an early clinical experience (a significant number of internships starting from the first semester of the third year) and a great attention to communication skills and relational aspects of patient-doctor relationship.

In the second year, a mandatory 1-week observational training experience at a general practitioner’s (GP) office is planned (16 h, 1 ECTS). This training exposes students to real-life applications of the bio-psycho-social model of medical conditions [47] and offers them a space to discuss the experience through debriefing activities. Clinical clerkship in hospital wards is outlined throughout the following academic years (about 400 h in year 3, 300 in year 4, 250 in year 5, and 500 in year 6) and provides about 50 ECTS. The formal and structured formative activities targeting communication skills and relational abilities are the second-year theoretical-practical course “Communication Skills” and the fifth-year course “Clinical Psychology.” The former course includes 24 h of frontal lessons, 12 h of practical classroom activities, and provides 3 ECTS. The latter is a 2 ECTS course and covers 12 h of frontal lessons and 12 h of practical classroom activities. Both courses are compulsory and equip students with theoretical knowledge and practical strategies on the interpersonal skills necessary to manage functional and effective doctor-patient encounters. In particular, the Communication Skills course provides students with a thorough understanding of the Calgary Cambridge model [48], the S.P.I.K.E.S. approach to break bad news [49], strategies for motivational interviews [50], the main theoretical models of empathy in medical settings [51], and hands-on activities (e.g., role-playing) on how to manage and regulate emotions emerging during doctor-patient interactions. The Clinical Psychology course introduces the basic notions of etiology and treatments in psychopathology and offers, through small group activities, the opportunity to reflect on the relational and communication aspects of their previous clinical clerkship.

### Procedure

This study was conducted across 7 years (from the academic year 2014/2015 to academic year 2020/2021) comprising four consecutive student cohorts entering the Medical School of the University of Milano-Bicocca in 2013, 2014, 2015, and 2016. Participants filled in paper-and-pencil questionnaires at the first semester of their second year of medical school (T0 — before GP’s office experience, clinical clerkship, and any academic communication skills and psychology courses) and the beginning of their fifth academic year (T1 — after GP’s office experience, 2-year clinical clerkship, and academic communication skills and psychology courses). At T0, students were approached and invited to take part to the study at the first scheduled class of “Communication Skills” while at T1 students were approached after the final scheduled class of “Clinical Psychology” by a researcher who was not one of their lectures.

The present study focused on the intertwined clinical and educational activities carried out between the beginning of year 2 to the beginning of year 5 which includes the “Communication Skills” course (year 2), GP’s office experience (year 2), clinical clerkship (from years 3 to 5), and “Clinical Psychology” course (year 5). These are the only formal activities that provide students with the opportunity to be exposed to and to reflect on the doctor-patient relationship during the study center medical school program. Participation was voluntary and no credits or monetary compensation were provided. All participants provided informed consent. This study was approved by the Ethical Committee of the University of Milano-Bicocca (protocol number: 39927).

### Measures

Self-reported questionnaires elicited information on socio-demographic characteristics and students’ identification numbers. The cognitive and emotional components of empathy were measured with the Italian version of the Jefferson Scale of Empathy – Student version (JSE-S) and the Interpersonal Reactivity Index (IRI).

The JSE-S [52, 53] is one of the most widely used questionnaires to detect empathy in medical education research [38]. This tool is based on the definition of empathy in the context of patient care as a predominantly cognitive attribute, which involves (a) the understanding of the patient’s experiences, concerns, and perspectives, and (b) the ability to communicate this understanding with the mere intention to be of help [54]. The JSE questionnaire includes 20 items rated on a 7-point Likert-type scale (1 = “I completely disagree”, 7 = “I completely agree”). JSE-S scoring ranges between 20 and 140. The JSE-S returns three levels of empathy: scores between 47 and 105 are considered as a low empathy level; 106 to 120 as a moderate empathy level; and 121 to 140 as a high empathy level [39]. In our sample, the internal consistency (Cronbach’s *α*) of the JSE-S total score was .86 at T0 and .87 at T1.

The IRI [55, 56] is a multidimensional questionnaire consisting of 28 items answered on a 5-point Likert scale (ranging from 0 = “does not describe me well” to 4 = “describes me very well”). It has four 7-item subscales: (a) Empathic Concern (IRI-EC), the other-oriented emotional dimension of empathy that evaluates the tendency of individuals to feel compassion, concern, and warmth towards other people who experience unpleasant experiences; (b) Personal Distress (IRI-PD), the self-oriented emotional domain of empathy which refers to the feelings of discomfort and anxiety experienced in tense interpersonal settings; (c) Perspective Taking (IRI-PT), cognitive domain of empathy which examines the ability to adopt the point of view of others; (d) Fantasy (IRI-F), cognitive domain of empathy which investigates the propensity to identify oneself with fictitious characters in books, cinema, or theater. Each IRI scale scoring ranges between 0 and 28. In our study, the Cronbach’s alpha was .78 for IRI-EC, .73 for IRI-PD, .79 for IRI-PT, and .85 for IRI-F at T0, whereas at T1 the reliability coefficients were .78 for IRI-EC, .83 for IRI-PD, .83 for IRI-PT, and .87 for IRI-F.

### Statistical Analyses

Possible differences between student cohorts were explored. A chi-square test of homogeneity (*χ*^2^) was used to determine whether the gender distributions were statistically significantly different in the four student cohorts. A set of analyses of variance (ANOVAs) was performed to test if there were significant differences in the study measures (JSE-S and IRI scores) and in age among the four student cohorts. Furthermore, a set of Student’s *t*-test was performed to explore differences between students who completed both the assessments and those who completed only T0.

Descriptive statistics for demographic data and empathy scores were calculated. Pearson’s correlations between JSE-S and IRI scores explored the statistical relationship between empathy measures. A set of ANOVAs for repeated-measures examined the statistical significance of changes in empathy dimensions at T0 and T1 while controlling for gender. Student’s *t*-tests explored gender differences in empathy scores at the two assessment points. We reported the effect size estimates eta-squared (*η*^2^) and Cohen’s *d* for the repeated-measures ANOVA and *t*-tests, respectively. Bonferroni correction was used for multiple comparisons and the significance cut-off was set at *p* < .025. All analyses were performed using the IBM SPSS Statistics version 24 for Mac.

## Results

### Study Participants and Descriptive Statistics

Four consecutive cohorts of Italian second-year medical students (*N* = 576) enrolled at the Medical School of the University of Milano-Bicocca were invited to participate in this study. Five hundred and thirteen students (response rate = 89.1%) agreed to participate and completed the assessment at T0 and 336 students (response rate = 65.5%) completed the second assessment at T1 with an attrition rate of 40% which fell into the range (from 30 to 70%) reported in several previous longitudinal studies [57]. Female participants were 278 (54.2%) at T0 and 194 (57.74%) at T1. The average age of participants at T0 was 20.03 years (SD = 1.41). All participants did not have children and were full-time students.

With regard to differences at T0 in demographics among the four student cohorts, the chi-square test and the ANOVA showed no statistically significant differences in gender distribution (*χ*^2^(3) = .774, *p* = .856) and age (*F*(3,572) = .566, *p* = .638), respectively. Moreover, ANOVAs showed that at T0 there were no differences in JSE-S and IRI scoring in the four student cohorts (*F*s(3,572) < 1.656, *p*s > .739). A set of *t*-tests indicated no statistically significant differences in empathy scoring at T0 between students who completed both assessments (T0 and T1) and those who completed only the T0 assessment (*t*s(574) < 1.728, *p*s > .085). Correlation coefficients between JSE-S total score and IRI dimensions are reported in Table 1. At both T0 and T1, the significant correlation coefficients between the two scales were positive.

**Table 1:** Pearson’s correlations for JSE-S and IRI dimensions

	1	2	3	4	5
1. JSE-S	–	.465**	−.001	.339**	.302**
2. IRI-EC	.458**	–	.143*	.368**	.334**
3. IRI-PD	**.079**	.156*	–	−.064	.223**
4. IRI-PT	.450**	.433**	−**.071**	–	.133*
5. IRI-F	.296**	.375**	.262**	.179**	–

Analyses above the diagonal are for T0; analyses below the diagonal (bold) are for T1

*JSE-S* Jefferson Scale of Empathy – Student version, *IRI-EC* Interpersonal Reactivity Index—Empathic Concern, *IRI-PD* Interpersonal Reactivity Index—Personal Distress, *IRI-PT* Interpersonal Reactivity Index—Perspective Taking, *IRI-F* Interpersonal Reactivity Index – Fantasy

*p < .025; **p < .001

### Longitudinal Changes

As reported in Table 2, the results of the ANOVAs for repeated-measures show that there was a statistically significant change in empathy dimensions between T0 and T1, except for the IRI-EC. At T1, medical students obtained higher scores on JSE-S, and IRI-PT, whereas IRI-PD and IRI-F scores significantly decreased. There was no statistically significant interaction between gender and year of study for all empathy dimensions (*F*s(1,334) < 3.573, *p*s > .060) which means that the pattern of change in empathy dimensions for men was similar to that of women. All differences had a small to medium effect size.

**Table 2:** Longitudinal change in empathy scores (JSE-S and IRI) controlled by gender from the second (T0) to the fifth (T1) year of study (*N* = 336)

	T0	T1			
	*M* (SD)	*M* (SD)	*F*	*p*	*η*^2^
JSE-S	112.76 (11.39)	115.61 (11.46)	22.869	< .001	.06
IRI-EC	18.87 (3.98)	19.25 (4.38)	3.005	.084	-
IRI-PD	9.86 (4.55)	9.19 (4.81)	6.125	.014	.02
IRI-PT	17.77 (4.41)	19.18 (4.47)	40.942	< .001	.11
IRI-F	18.11 (5.43)	17.38 (5.77)	8.475	.004	.03

*JSE-S* Jefferson Scale of Empathy – Student version, *IRI-EC* Interpersonal Reactivity Index—Empathic Concern, *IRI-PD* Interpersonal Reactivity Index—Personal Distress, *IRI-PT* Interpersonal Reactivity Index—Perspective Taking, *IRI-F* Interpersonal Reactivity Index—Fantasy

### Gender Differences

As shown in Table 3, gender differences at the two assessment points were statistically significant for all empathy dimensions (JSE-S and IRI), and women outscored men in all the empathy scales at both T0 and T1. All differences had a small to medium effect size.

**Table 3:** Gender differences in empathy scores (JSE-S and IRI) in second (T0) and fifth (T1) year of study

	T0 (*N* = 513)					T1 (*N* = 336)				
	Male (*N* = 235)	Female (*N* = 278)				Male (*N* = 142)	Female (*N* = 194)			
	*M* (SD)	*M* (SD)	*t*	*p*	*d*	*M* (SD)	*M* (SD)	*t*	*p*	*d*
JSE-S	109.08 (12.85)	114.11 (10.83)	−4.808	< .001	.42	112.15 (12.33)	118.12 (10.09)	−4.887	< .001	.53
IRI-EC	17.36 (4.36)	19.52 (3.81)	−5.976	< .001	.53	18.01 (4.40)	20.16 (4.13)	−4.587	< .001	.50
IRI-PD	8.63 (4.27)	10.42 (4.67)	−4.498	< .001	.40	8.46 (4.57)	9.73 (4.93)	−2.404	.017	.27
IRI-PT	17.16 (4.57)	18.10 (4.38)	−2.363	.019	.21	18.55 (4.77)	19.64 (4.19)	−2.231	.025	.24
IRI-F	16.28 (5.64)	18.74 (5.47)	−5.011	< .001	.44	15.91 (5.61)	18.45 (5.65)	−4.081	< .001	.45

*JSE-S* Jefferson Scale of Empathy – Student version, *IRI-EC*, Interpersonal Reactivity Index—Empathic Concern, *IRI-PD* Interpersonal Reactivity Index—Personal Distress, *IRI-PT* Interpersonal Reactivity Index—Perspective Taking, *IRI-F* Interpersonal Reactivity Index—Fantasy

## Discussion

This is the first longitudinal study considering both emotional and cognitive dimensions of empathy in Italian undergraduate medical students. We identified a significant association between empathy and a medical school program encompassing clinical clerkship and academic communication skills courses with no effect of gender confirming our hypothesis that medical students’ empathy would change as medical studies progress. When looking at the two components of empathy, cognitive empathy resulted to increase more throughout medical school than the emotional component which seems to benefit less from the medical school curriculum. We furthermore confirmed that female students would report higher emotional and cognitive empathy than male students throughout the medical education path.

### Changes in Empathy Throughout Medical School

In medical education, hospital-based internships and exposure to patients foster the acquisition of both clinical and relational abilities, which are essential skills for building the therapeutic alliance. Empathy is a key element in this process: knowing the trajectory of its changes is essential to better planning curriculum. We observed that medical students after 2 years of clinical clerkship and the formative activities targeting communication skills and relational abilities entailed in the study showed (a) greater capability to understand and consider patients’/others’ experiences, concerns, and perspectives (cognitive side of empathy assessed by JSE-S and IRI-PT scales); (b) less emotional distress in tense interpersonal settings (IRI-PD); (c) a decreased tendency to imaginatively transpose themselves into fictional characters and situations (IRI-F); and (d) no significant change in concern for the unfortunate of others (IRI-EC).

Although some studies reported increases in cognitive empathy during medical school [58, 59], the increase in the cognitive empathy indexes (JSE-S and IRI-PT) in our sample is not in line with previous studies which observed a decline in the cognitive empathy in healthcare student samples [28, 60–62]. Echoing other scholars [63, 64], we posit that the massive exposure to the clinical encounters during clinical clerkship may have increased the importance that medical students give to taking others’ viewpoints. Additionally, the characteristics of the curriculum of the medical school in which the study took place can help us to further explain these results. The importance that the university study center gives to communication skills and doctor-patient relationships may have promoted and strengthened an empathic other-oriented attitude in medical students. The two mandatory theoretical-practical courses, namely the second-year “Communication Skills” and the fifth-year “Clinical Psychology” courses, provided students with the opportunity to reflect on the emotional/relational implications of the medical profession and on the interpersonal skills required to manage doctor-patient relationships effectively. The attention to the interpersonal and communication dimensions given by the study center medical curriculum may have mitigated the well-documented factors that have been proven to hamper empathy in healthcare providers and students, including the “hidden curriculum” and negative role models, unrealistic expectations and loss of idealism, and pressing educational demands [28, 36, 60, 65, 66]. However, we can only speculate and no conclusive considerations can be drawn about the role of the study center medical curriculum on our findings as a comparison with medical programs lacking a formal investment in communication skills is needed to test this hypothesis.

The decrease in the IRI-PD scores in our sample is consistent with Paro et al.’s study [67] where medical students reported lower IRI-PD scores at the end of the medical training than those exhibited in their first year of studies. Having attended clinical situations characterized by interpersonal suffering for a prolonged period could have promoted greater emotional regulation and a greater sense of self-efficacy in dealing with tense clinical settings [20, 68]. Furthermore, the practical activities targeting emotions emerging during doctor-patient interactions as part of the academic courses might have bolstered emotion regulation abilities in our sample. This result could be considered a desirable outcome of the medical education process since high levels of distress and anxiety in tense and emergency settings could be detrimental to professional performance in healthcare delivery [69].

Although the IRI-F scale has shown a positive correlation with other cognitive empathy dimensions (in line with existing data [70]), its trajectory throughout medical school was opposite to them (i.e., JSE-S and IRI-PT). It might not be surprising that medical students decrease in IRI-F as their studies progressed. Previous studies identified a positive association between IRI-F and emotional vulnerability [71], sensitivity to others [2], and difficulties in controlling emotional reactions (72). Throughout medical school, students were provided with structured interpersonal skills training and were heavily exposed to patients’ and their families’ emotions during clinical clerkships. These activities may have increased emotional self-regulation and promoted emotional detachment from tense professional situations (as supported by the decrease in IRI-PD in our sample) leading to a decrease in fantasy orientation as a coping strategy to interpersonal distress (i.e., IRI-F). As no study has directly explored the relationship between IRI-F and year of medical study, further research is needed to deepen our understanding of this association.

In line with existing data [42, 73–75], we found no significant change in concern for unfortunate others (IRI-EC) throughout medical school. IRI-EC was the IRI dimension that received the highest scores both at T0 and at T1 (18.87 and 19.30 out of 28 points, respectively). This emotional component of empathy may not have significantly changed because it may reflect one of the strongest drives for entering medical school, namely, the motivation to help people in need [76]. The medical school program of the study center seems to sustain this motivation for a medical career.

### Gender Differences in Empathy

Stereotypical differences between personal values profiles held by males and females have been advanced to explain gender differences in empathy [27, 77]. In our Western-industrial culture, these gender stereotypical profiles direct women to exhibit and cultivate self-transcending and other-oriented values while leading men to focus on self-enhancing values such as personal power and achievement. Evolutionary gender differences have been also offered to explain why females exhibit higher levels of care and love [78]. Rueckert and Naybar [79] reported a positive correlation between right hemisphere activation on a face recognition task and empathy in women but not in men suggesting a possible neural basis for gender differences in empathy.

### Strengths and Limitations

This is the first study in an Italian setting that adopted a longitudinal research design to describe the trajectory of undergraduate medical students’ empathy, and the impact of gender over time. This study employed validated widely used measures to examine medical students’ empathy enhancing cross-national and cross-cultural comparisons. There are several limitations to our study. Since this was a single-institution study, the generalizability of our results must be done with caution. Although study participants were nearly 60% of the study medical school students, our findings may not be representative of the Italian medical student population. Another limitation of this study is the lack of a control group: non-medical students and students enrolled at medical schools with no formal or structured formative activities targeting communication skills and relational abilities could have enabled comparisons. Future studies may supplement our results and overcome our limitations including control groups and considering actual behaviors in clinical settings. Furthermore, the inclusion of an observational assessment of empathy (e.g., simulated or real patients with external raters) could improve the ecological validity of future investigations. It could be also advisable to include more assessment points along the academic path to further capture the contribution of each formal and structured formative activity in promoting empathy growth.

## Conclusion

Our findings support the idea that medical students, as their studies progress, are more prone to take care of patients’ emotional needs, consider others’ viewpoints, and experience less anxiety in tense interpersonal settings. Female medical students exhibited higher emotional and cognitive empathy than their male counterparts throughout medical school. Our findings suggest that the medical curriculum may impact undergraduate medical students’ empathic attitudes. Medical administrators and educators should consider incorporating class interventions into the pre-clinical and clinical curriculum to allow students to reflect on the emotional and relational implications of the medical profession before and after the clinical clerkship. Those interventions could include methodologies that have been proven to enhance and sustain empathy such as patient narrative and creative arts, writing and drama, communication skills training, problem-based learning, patient interviews, and experiential learning (80–82). Further research is needed to deepen the understanding of educational factors that foster positive changes in cognitive empathy we observed in our sample.

## Data Availability

The datasets generated during and/or analyzed during the current study are available from the corresponding author on reasonable request.
